# ENGAGEMENT AND EFFECTIVENESS OF USING MOBILE APP FOR DIABETES SELF-MANAGEMENT AMONG OLDER ADULTS

**DOI:** 10.1093/geroni/igz038.2904

**Published:** 2019-11-08

**Authors:** Mei-Lan Chen, Shinyi Wu, Pey-Jiuan Lee, Haomiao Jin

**Affiliations:** 1 Byrdine F. Lewis College of Nursing and Health Professions, Georgia State University, Atlanta, Georgia, United States; 2 University of Southern California, Los Angeles, California, United States; 3 Suzanne Dworak-Peck School of Social Work, University of Southern California, Los Angeles, California, United States

## Abstract

Mobile health applications (app) have shown to be beneficial for chronic disease management. However, few studies assessed older adults’ engagement in tracking self-management activities with app functions and effectiveness on improving their diabetes outcomes. This study investigated tracking patterns of each app function (blood sugar, blood pressure, diet, exercise, medication adherence etc.) in a graphic-based aging-friendly diabetes self-management app (IMTOP app) and associated the patterns with changes in HbA1c, self-care behavior, diabetes empowerment, and health promotion. The sample included 334 community-dwelling older adults with type 2 diabetes in Taiwan (mean age 64.57 ± 6.64 years) participated in the IMTOP training course that designed to motivate and train older adults with diabetes to use mobile tablets and apps. We performed trajectory analyses using SAS TRAJ procedure to identify distinct classes of individuals following similar longitudinal patterns on absence or presence of weekly app use for each individual app function. The relationships between the app engagement class memberships and 4- and 8-month diabetes health outcomes were assessed using an econometric regression analysis approach. The results showed the degree of app engagement on any single function was significantly and positively correlated with diabetes self-care scale scores (all p < .05). Only the engagement on the blood sugar function had statistically significant association with HbA1c improvements (p < .05). The app use was not associated with diabetes empowerment or health promotion. The study findings suggest any app function engagement significantly improved older adults’ overall self-management but blood sugar tracking is critical to improve HbA1c.

